# Water-Permeable Dialysis Membranes for Multi-Layered Microdialysis System

**DOI:** 10.3389/fbioe.2015.00070

**Published:** 2015-06-02

**Authors:** Naoya To, Ippei Sanada, Hikaru Ito, Gunawan S. Prihandana, Shinya Morita, Yoshihiko Kanno, Norihisa Miki

**Affiliations:** ^1^Department of Mechanical Engineering, Keio University, Yokohama, Japan; ^2^Department of Urology, Keio University School of Medicine, Tokyo, Japan; ^3^Department of Nephrology, Tokyo Medical University, Tokyo, Japan

**Keywords:** water permeable, dialysis, membrane, hemofiltration, micro, implantable, artificial kidney, polyethersulfone

## Abstract

This paper presents the development of water-permeable dialysis membranes that are suitable for an implantable microdialysis system that does not use dialysis fluid. We developed a microdialysis system integrating microfluidic channels and nanoporous filtering membranes made of polyethersulfone (PES), aiming at a fully implantable system that drastically improves the quality of life of patients. Simplicity of the total system is crucial for the implantable dialysis system, where the pumps and storage tanks for the dialysis fluid pose problems. Hence, we focus on hemofiltration, which does not require the dialysis fluid but water-permeable membranes. We investigated the water permeability of the PES membrane with respect to the concentrations of the PES, the additives, and the solvents in the casting solution. Sufficiently, water-permeable membranes were found through *in vitro* experiments using whole bovine blood. The filtrate was verified to have the concentrations of low-molecular-weight molecules, such as sodium, potassium, urea, and creatinine, while proteins, such as albumin, were successfully blocked by the membrane. We conducted *in vivo* experiments using rats, where the system was connected to the femoral artery and jugular vein. The filtrate was successfully collected without any leakage of blood inside the system and it did not contain albumin but low-molecular-weight molecules whose concentrations were identical to those of the blood. The rat model with renal failure showed 100% increase of creatinine in 5 h, while rats connected to the system showed only a 7.4% increase, which verified the effectiveness of the proposed microdialysis system.

## Introduction

Dialysis therapy is well developed and widely used for patients with end-stage renal diseases, but the patients must undergo this treatment in hospital for 4 h, three times a week (Kanno and Miki, 2012). Implantable dialysis systems would reduce the frequency of hospital visits and drastically improve their quality of life (Yang et al., 2002; Xu and Qusay, 2004; Gu and Miki, 2009). In addition, the treatment can be conducted for 24 h, 7 days/week, whereas the dialysis therapy runs only for 4 h, 3 days/week. The rather slow and gentle treatment is less harmful to the patients.

The dialysis membrane is one of the critical components that determine dialysis performance. These membranes allow only low-molecular-weight molecules, such as sodium, potassium, urea, and creatinine, to pass through while blocking proteins, such as albumin, and other larger molecules. Commercialized dialyzers have membranes made of nanoporous polymers, which include cellulose, cellulose acetate, polyethersulfone (PES), polyamide, and poly(methyl methacrylate) (Sakai, 1994). Micro/nanofabricated silicon membranes have been proposed and tested as the dialysis membranes of implantable systems (Fissell and Roy, 2009). The pore sizes, distribution, and even location can be precisely controlled. Other candidates for the porous membranes include glass, ceramics, polymers, and metals, such as platinum black (Tsuru, 2001; Cruz et al., 2005; Grigoras et al., 2005; Black et al., 2006; Boughaba and Wang, 2006; Shinohara et al., 2006). Our group proposed nanoporous membranes made of PES (Gu and Miki, 2007). The PES membranes are formed by the phase inversion method, where the casting solution can adjust the permeability of the molecules with respect to the sizes (Gu and Miki, 2007). The PES membranes were sandwiched by microchannels to form the microdialysis system, which was experimentally verified to have efficiency as high as a healthy kidney. Long-term stability of the PES membranes was investigated in *in vitro* experiments using whole cow blood, where nanoporous parylene (Prihandana et al., 2012, 2014a) and fluorinated diamond-like carbon (Prihandana et al., 2013) were deposited to modify the surface properties of the membranes.

**Figure 1:**
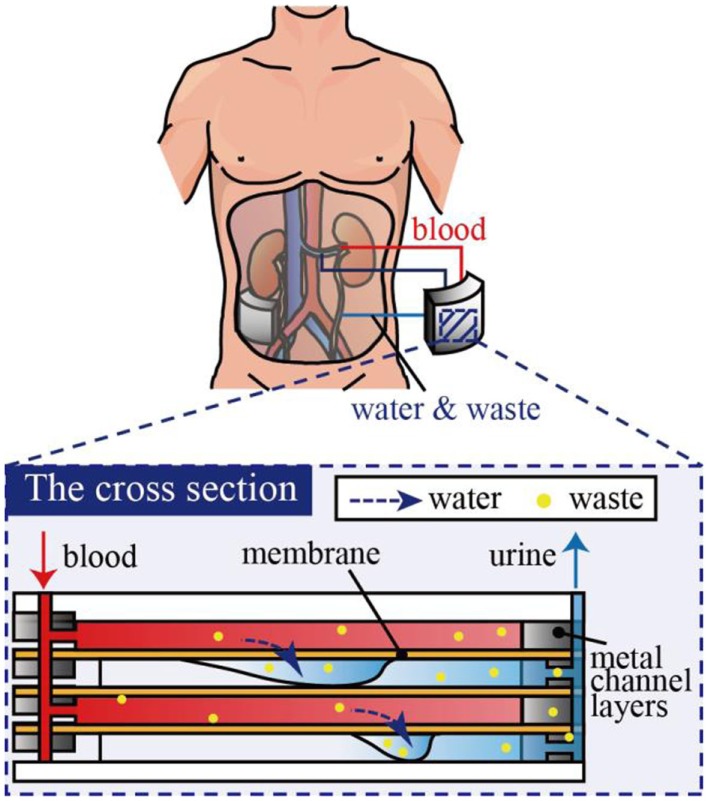
**Concept of no-dialyzate microdialysis system for implantable artificial kidney**.

Another challenge for implantable dialysis systems is miniaturization of the whole system. In our previously proposed system, microchannels were designed such that blood could be introduced at a sufficiently high-flow rate via blood pressure, i.e., no pump was necessary to drive the blood. However, dialysis fluids need to be stored, introduced, and cleaned, which hinders miniaturization of the system. Therefore, we consider that hemofiltration is advantageous over hemodialysis since hemofiltration does not use dialysis fluid, but removes low-molecular-weight molecules from blood as an aqueous solution, as shown in Figure 1 (Jeffrey et al., 1994). While this may lead to a lack of necessary low-molecular-weight molecules, these can be later supplied by medication and/or diet.

For hemofiltration, the membranes have to possess sufficient water permeability while blocking proteins. The PES membrane used in prior work did not exhibit sufficient water permeability (Gu and Miki, 2007). In this paper, we investigate the water permeability of the membrane with respect to the concentrations of PES, additives, and solvents of the PES casting solution. First, we conducted *in vitro* experiments using whole cow blood to find the best casting solution with respect to the water permeability, diffusion capacity of low-molecular-weight molecules, blocking of proteins, and mechanical stability. Then, we characterized the membrane in *in vivo* experiments using nephrectomized rats.

## Materials and Methods

### Materials

Polyethersulfone (molecular weight of 4800, Sumitomo Chemical Co., Japan) was used as a solute for the membrane. Polyethyleneglycol (PEG, molecular weight of 1000, Wako Pure Chemical Industries, Ltd., Japan) and *N*,*N*-dimethylacetamide (DMAc, Wako Pure Chemical Industries, Ltd., Japan) were used as the additive and solvent in the PES casting solution, respectively. An SUS316L plate 200 μm in thickness was purchased from Nilaco Co., Japan. SU-8 3050 (MicroChem Co., Japan) was used as a photoresist for photolithography.

Defibrinated bovine blood used in *in vitro* experiments was purchased from Kojin Bio Co., Ltd. Sodium chloride, urea, and potassium chloride (KCl) were purchased from Wako Pure Chemical Industries, Ltd., Japan. Saline, heparin, and 32- to 36-week-old Sprague-Dawley rats were used in *in vivo* experiments and were purchased from Otsuka Pharmaceutical Factory Co., Japan, Mochida Pharmaceutical Co., Ltd., Japan, and Clea Japan, Co., Japan, respectively. Isoflurane purchased from Wako Pure Chemical Industries, Ltd., Japan was used as the anesthesia. Ethanol and 20% glutaraldehyde solution were purchased from Wako Pure Chemical Industries, Ltd., Japan.

### PES membrane formation

Polyethersulfone membranes were formed from a casting solution composed of PES, PEG, and DMAc. The investigated mixing ratios of the solute, additive, and solvent are described in Table 1. In this experiment, we set the ratio of PES and PEG constant, which was reported to be a suitable ratio (Chong et al., 2012). The casting solution was stored at room temperature for 1 day after being mixed to become transparent. Subsequently, the PES casting solution was poured onto a glass substrate and spin-coated at 300 rpm to have a thickness of 100 μm. The glass substrate was then immersed into deionized (DI) water for 1 day at room temperature to remove PEG from the membrane. The PES membrane was gelatinized and became a white nanoporous membrane. The pore sizes cannot be directly measured by SEM since they are too small. However, they are considered to be on the order of nanometers, which allows low-molecular-weight molecules to pass through and blocks large molecules, such as albumin.

**Table 1:** **Mixing ratio (wt%) of PES/PEG/DMAc and their structures**.

	PES	PEG	DMAc	
1	10.0	8.3	81.7	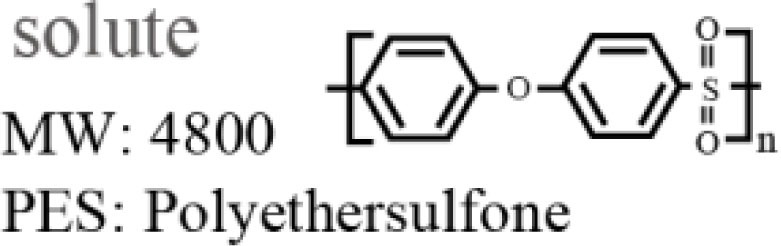
2	12.5	10.4	77.1	

3	15.0	12.5	72.5	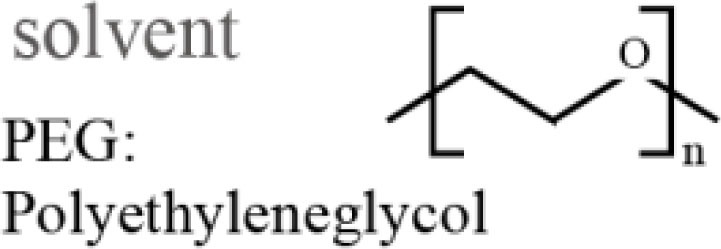
4	17.5	14.5	68.0	

5	20.0	16.7	63.3	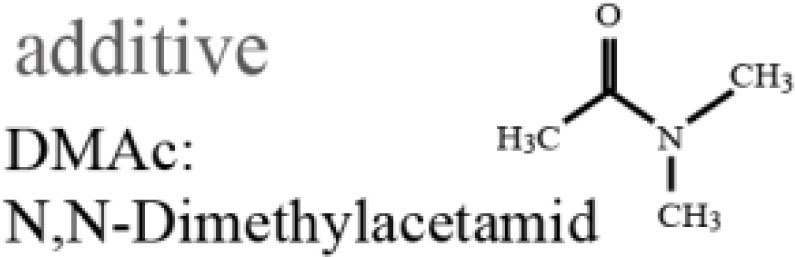
6	22.5	18.8	58.7	
7	25.0	20.8	54.2	

### Fabrication of microdialysis devices

**Figure 2:**
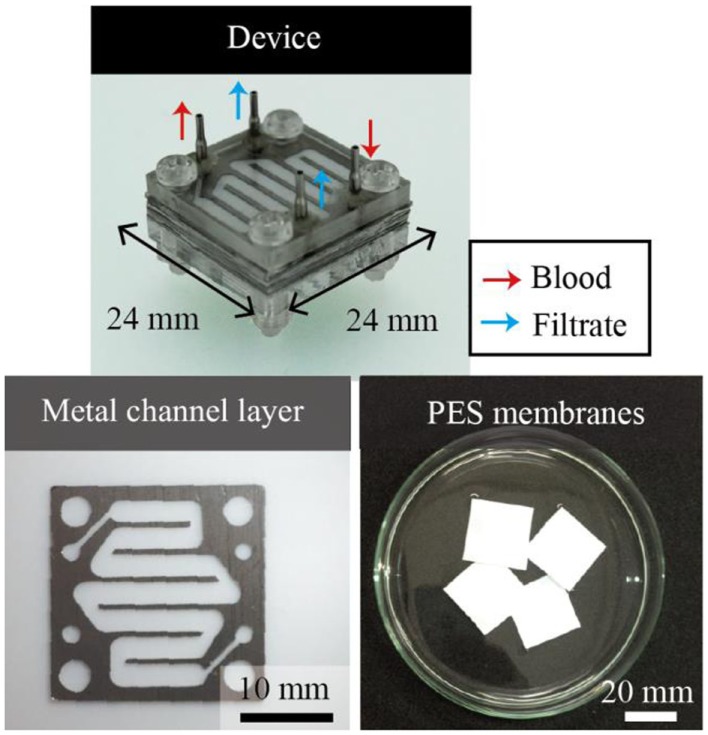
**Multi-layered microdialysis device consisting of PES membranes and metal channel layers**. The ports are used as the inlet and outlet for blood and to access the filtrates. The metal channel layers are formed by electrolytic etching. PES membranes and metal channel layers are stacked alternately. The channels are connected in parallel, which allows the total mass flow to be increased without increasing the required pressure.

The microdialysis system tested in this work comprised multiple sets of a porous PES membrane and two microfluidic channels made of SUS316L that sandwich the membrane, as shown in Figure 2. The system has four ports: the outlet and inlet for the blood and two access ports for the filtrate. The wide channels are 2 mm in width and 72 mm in total length. The channel height, or the thickness of the SUS316L plate, is 200 μm. The channels are designed to have a sufficient volumetric flow rate at a human blood pressure (Gu and Miki, 2009) (see Figure S1 in Supplementary Material). SUS316L is a highly biocompatible material. The microchannels are connected in parallel such that the total volume of blood can be increased with the number of layers without increasing the pressure.

The metal layers were formed by electrolytic etching with photolithographically patterned SU-8 3050 as the masking material. The electrolyte consists of ethylene glycol, sodium chloride, citric acid, and DI water. The electrolytic etching was conducted at an applied voltage of 20 V for 5 h. The etching setup is shown in Figure S2 in Supplementary Material. We overlaid the PES membranes and the metal layers, and the alignment holes and screw holes were punched through the PES membranes with a disposable biopsy punch (Kai Co., Japan). The PES and metal layers were bonded using the DMAc solvent as the glue.

### *In vitro* experiments

First, we deduced the most suitable PES membrane for the no-dialyzate microdialysis system among those formed from the PES casting solutions listed in Table 1. Whole cow blood was introduced into the single-layered device, which consisted of two microchannel layers and a PES membrane, using a syringe pump at a flow rate of 100 ml/min for 100 min, when the pressure of 200 mmHg was applied (see Figure S3A in Supplementary Material for the setup). First, the membrane was optically inspected to assess the mechanical stability. Fractures in the membrane allow blood to leak through the membrane. The filtrate was collected and investigated with respect to the water permeability, diffusion capacity of low-molecular-weight molecules, and blocking of proteins.

Water permeability of the membrane was characterized by the filtration coefficient *L*_P_, which is defined as Eq. 1.

(1) $$L_{P}=\frac{V_{\text{F}}}{T_{\text{F}}\times \mathrm{TMP}\times A}$$

where *V*_F_ is the volume of the permeated water (milliliters), *T*_F_ is the filtration time (minutes), *TMP* is the pressure difference between the inlet and outlet of the membrane (millimeter of mercury), and *A* is the area of the membrane (square meter). *TMP* was measured using the following Eq. 2.

(2) TMP=ΔP−Δπ

where ΔP is the pressure difference of blood between the inlet and outlet (millimeter of mercury) and Δπ is the osmotic pressure caused by protein in blood (millimeter of mercury), which is defined as Eq. 3.

(3) $$\Delta \pi =C_{\text{A}}\mathrm{iRT}$$

where *C*_A_ is the molar concentration of the protein (mole per liter), *i* is a dimensionless number (−), *R* is the gas constant [0.0083 l⋅MPa/(mol⋅K)], and *T* is the absolute temperature (Kelvin). The target value of the filtration coefficient *L*_P_ to meet the requirements for hemofiltration treatment of humans was obtained from a medical doctor; the volume of filtrates needs to be equivalent to that of urine (~1500 ml for adults) at the human blood pressure.

Next, using the best PES membrane, we investigated how the number of layers affected the total volume of the filtrate. Scale-up of the system, in this case by increasing the number of layers, is crucial for practical applications. We prepared single-layered and multi-layered devices that had 1, 5, and 10 layers, respectively. Since the syringe pump could not supply a sufficient amount of cow blood to the multi-layered device, we used a peristaltic pump to circulate the cow blood (see Figure S3B in Supplementary Material for the setup). The inlet pressure was set to be 130 mmHg. The filtration coefficient *L*_P_ was deduced for each device.

Concentrations of sodium, potassium, chloride, blood urea nitrogen (BUN), and albumin were measured using a POCT Automated analyzer for clinical chemistry (SPOTCHEM EZ, ARKRAY Inc., Japan). These concentrations were compared to those of the whole cow blood.

### *In vivo* experiments

**Figure 3:**
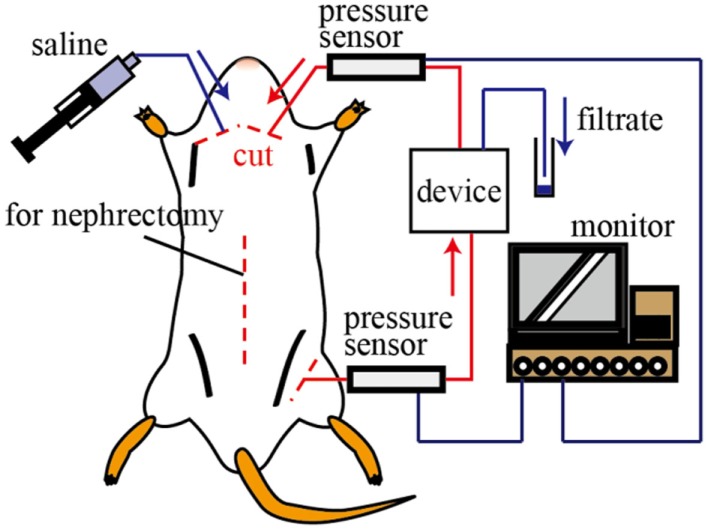
**Schematic illustration of the *in vivo* test**. The inlet and outlet of the device were connected to the femoral artery and jugular vein, respectively. The blood pressure of the rat was monitored during the experiments. The volume and components of the filtrate were investigated.

All animals used in the experiments received humane care. The experimental protocol was approved by the Laboratory Animal Care and Use Committee following Keio University guidelines. Female Sprague-Dawley rats (age, 32–36 weeks; Clea Japan, Co., Japan) were anesthetized with isoflurane and their limbs were fixed to the surgical table with adhesive tape. The abdominal cavity was opened and the renal function was halted by ligating the renal artery to stop the blood flow into the kidney. Medical tubes 0.58 and 0.965 mm in inner and outer diameters, respectively, were connected to the femoral artery and jugular vein, which were sequentially connected to the device inlet and outlet, as shown in Figure 3. The device and the tubes were filled with heparin solution (heparin:saline = 1:9) in advance. The blood pressure was monitored by a polygraph (RMT 1000, Nihon Koden, Co., Japan) during the experiments.

We tested 1, 5, and 10 layer devices. Three experiments were conducted for each device. After 30 min, the device was connected to the rat and the circulating system was formed, we collected the filtrate every 15 min for 80 min. After collecting the filtrate, we injected 5 ml of saline to recover the blood pressure. The volume of the filtrate was measured to evaluate the water permeability by the filtrate coefficient, and the concentrations of solutes were measured using the POCT Automated analyzer.

To verify the effectiveness of the hemofiltration treatment with the device, we investigated the increase of creatinine in the blood of nephrectomized rats with and without the device. Experiments were repeated three times. Creatinine is produced by metabolic reaction, and changes in creatinine levels are widely used to evaluate renal function. The concentration of creatinine increases when renal function is disrupted. The concentration of creatinine was measured for 7 h using the POCT Automated analyzer. For the rat with the device, the first 2 h was used to connect the device to the rat, and the treatment was conducted for 5 h.

## Results and Discussion

### Water permeability of the PES membrane

**Figure 4:**
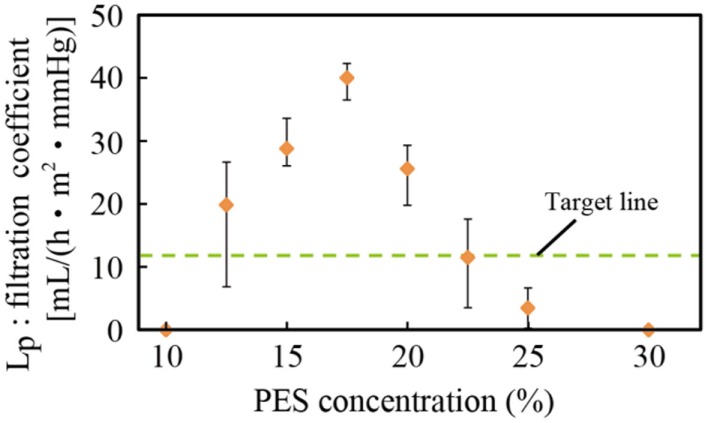
**Relationship between PES concentration and the filtration coefficient**. A PES concentration of 17.5% was experimentally found to have the best filtration coefficient and sufficient mechanical strength.

The relationship between the PES concentration of the casting solution and the filtration coefficient *L*_P_ is shown in Figure 4. Three samples were tested, and the error bars represent the minimum and maximum. The target line was drawn based on a value obtained from a medical doctor to meet the requirements for hemofiltration treatment of humans. The PES membrane formed from the casting solution with a PES concentration of 17.5% had the highest filtration coefficient among all the membranes. It possessed water permeability higher than the target value and was also experimentally verified to have sufficient mechanical strength. Thus, this membrane was used in the subsequent experiments.

There was less PEG additive in membranes with low concentrations of PES, and these membranes were considered to have fewer pores. Therefore, the water permeability increased with the PES concentration. By contrast, when the PES concentration was high, the thickness of the nanoporous skin layer of PES, which determined the permeability, was large, resulting in low water permeability. This has been reported in previous work (Chong et al., 2012).

### Filtration capacity of multi-layered devices

**Figure 5:**
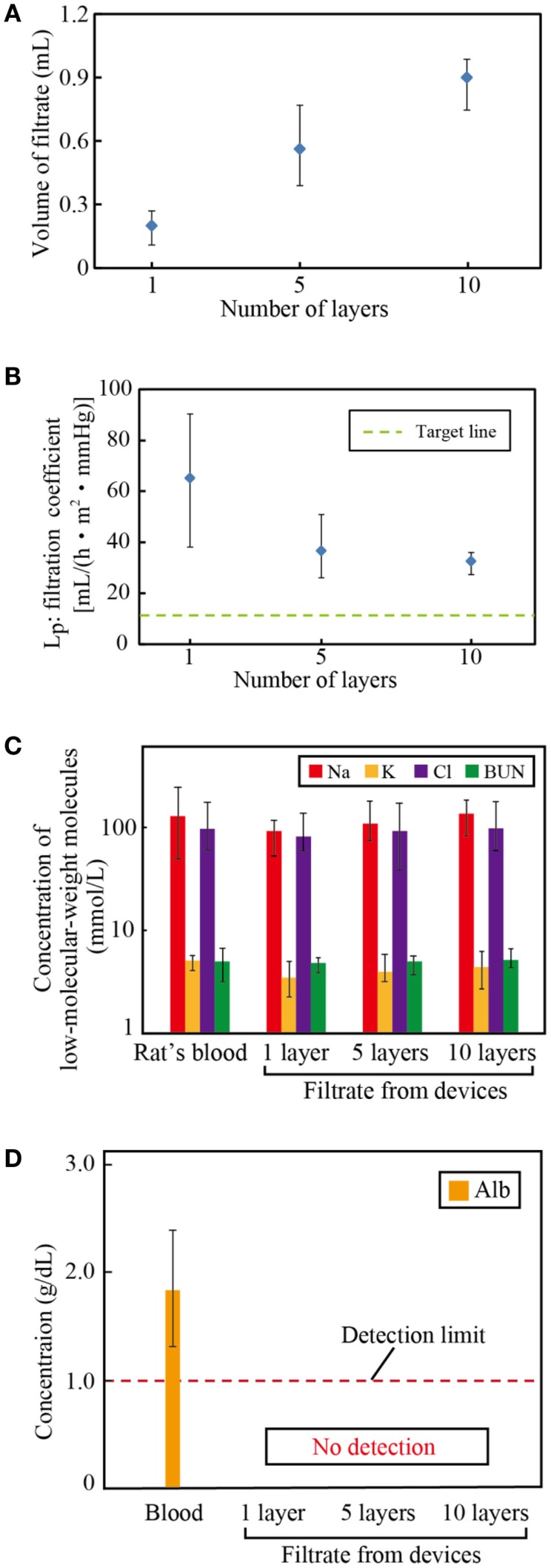
**Filtration capacity of the multi-layered devices**. **(A)** Volume of filtrate, **(B)** the filtration coefficient, and the concentrations of **(C)** low-molecular-weight molecules: sodium (Na), potassium (K), chlorine (Cl), and blood urea nitrogen (BUN) and **(D)** albumin (Alb) in the filtrate. The volume of filtrate increases with the number of layers, which verifies that the system can be scaled-up to satisfy the requirements for humans. The filtrate has almost identical concentrations of solutes as the blood, while it contains no albumin.

We assembled 1, 5, and 10 layer devices using PES membranes formed from 17.5% PES casting solution, which was found to have the best water permeability, as described in Section “Water Permeability of the PES Membrane.” Figures 5A,B show the volume of filtrate from the multi-layered device and the resulting *L*_P_. Three samples were tested, and the error bars represent the minimum and maximum. Results indicate that the system can be scaled-up by increasing the number of layers. The slight decrease of *L*_P_ with the layer number originates from the pressure drop at the inlet of the device.

Figures 5C,D show the concentrations of low-molecular-weight molecules and albumin of the blood and the filtrate, respectively. Three samples were tested, and the error bars represent the minimum and maximum. The concentrations of sodium, potassium, chloride, and BUN of the filtrate are almost identical to those of the blood. By contrast, albumin, the smallest protein in the blood plasma, was successfully blocked in all devices. These filtration properties satisfy the requirements for hemofiltration.

### *In vivo* tests

After the device was connected to the rat, the blood pressure dropped and then fluctuated drastically. This is because a fair amount of blood was extracted from the body to fill the tubes and devices, which was compensated by injecting saline. After 1–2 h, the blood pressure stabilized (see Figure S4 in Supplementary Material).

**Figure 6:**
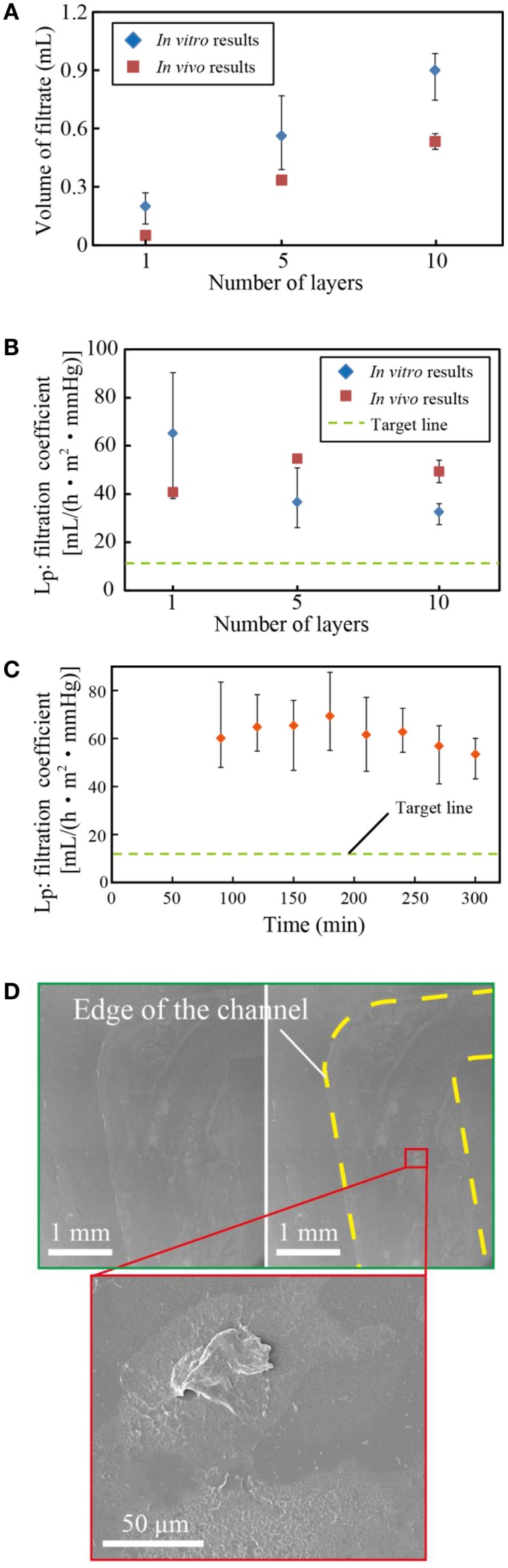
**(A)** Volume of filtrate from multi-layered microfilters and *in vivo* tests. **(B)** The filtration coefficient, and **(C)** the filtration coefficient of the devices for 5 h. **(D)** SEM photo of the tested membrane. The volume of filtrates in *in vivo* tests was slightly smaller than that in *in vitro* tests due to the lower blood pressure, while the filtration coefficients were comparable. The coefficient was almost constant with respect to the test periods, although some biomaterials were found on the PES membrane.

Figure 6A shows the volume of filtrate from our devices in *in vitro* and *in vivo* tests. The volume of filtrate was beyond our target in both *in vitro* and *in vivo* tests. The discrepancy between the volume of filtrates in *in vitro* and *in vivo* tests originates from the difference in the tension membrane pressure: 130 mmHg *in vitro* and 50–80 mmHg *in vivo*. As shown in Figure 6B, the filtration coefficients were comparable. The filtration coefficient remained almost constant over 5 h of experiments as shown in Figure 6C. Once experiments were completed, we investigated the membranes using scanning electron microscopy after osmium coating. As shown in Figure 6D, fibrotic tissues were observed on the surface. In our previous work, we conducted long-term *in vitro* diffusion tests for 28 days using bare PES and surface-treated PES membranes (Prihandana et al., 2013, 2014a). The diffusion capacity of the bare PES membrane deteriorated in the first 7 days; however, it stabilized for the rest of the test periods. In addition, we experimentally confirmed that the fibrotic tissues formed on porous membranes allow low-molecular-weight molecules to permeate the membranes (Prihandana et al., 2014b). These results indicate that the dialysis membrane can maintain the diffusion capacity even with the presence of fibrotic tissues. Therefore, we consider this formation is acceptable, though long-term experiments still need to be conducted to verify it. However, because the long-term experiments performed by opening the abdomen of rats were challenging, we will conduct future experiments using larger animals.

**Figure 7:**
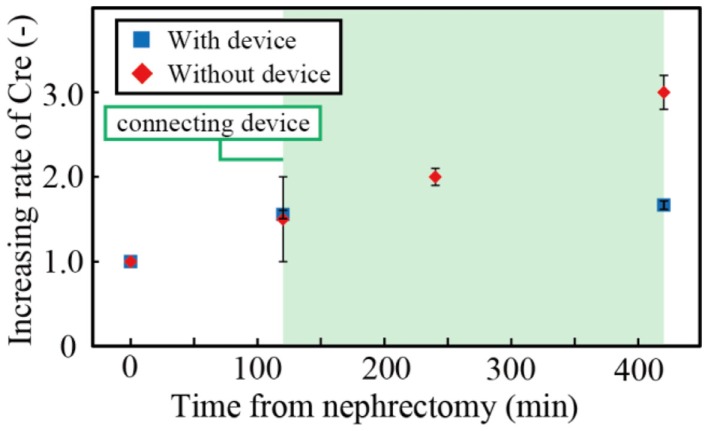
**Concentration of creatinine (Cre) in the nephrectomized rats with or without our device**.

We investigated the concentrations of creatinine in the blood after nephrectomy. Figure 7 shows the creatinine concentration with and without the filtration device. Three samples were tested, and the error bars in the figure represent the minimum and maximum. The creatinine concentration was normalized by that when the renal function was halted. The operation to connect the device to the rat took 2 h. During the subsequent 5 h, the creatinine concentration of the rat without the device increased by ~100%, while that of the rat connected to the device increased by only 7.4%. The results indicated that the developed filtration device could suppress the increase of creatinine and, with an appropriate scale-up of the system, it can replicate renal function sufficiently.

## Conclusion

We experimentally deduced the most appropriate PES casting solution to form a sufficiently water-permeable PES membrane. The membrane was verified to have a good diffusion capacity for low-molecular-weight molecules, while it could block albumin completely. Scale-up of the system was achieved by testing multiple layers of the PES membrane sandwiched by microchannel layers in both *in vitro* and *in vivo* experiments with rats. The filtration system could successfully suppress the increase of creatinine of a rat by 92.6% after 5 h of treatment. These results verified that this PES membrane had sufficient water permeability for an implantable microdialysis system.

## Conflict of Interest Statement

The authors declare that the research was conducted in the absence of any commercial or financial relationships that could be construed as a potential conflict of interest.

## Supplementary Material

The Supplementary Material for this article can be found online at http://journal.frontiersin.org/article/10.3389/fbioe.2015.00070/abstract
