# Supplementary material for: Water-Permeable Dialysis Membranes for Multi-Layered Microdialysis System
Source: Front Bioeng Biotechnol. 2015 Jun 2;3:70. doi: 10.3389/fbioe.2015.00070 (PMC4451643; doi:10.3389/fbioe.2015.00070)
Supplement: Supplementary file 1 [file Data_Sheet_1.PDF]

Supplementary Material

Water-Permeable Dialysis Membranes for Multi-Layered Micro Dialysis System

***Naoya To[1], *Ippei Sanada[1], Hikaru Ito[1], Gunawan S. Prihandana[1], Shinya Morita[2], Yoshihiko Kanno[3] and Norihisa Miki[1] (* Equal contribution)**

[1]Department of Mechanical Engineering, Keio University, Yokohama, Japan
[2]Department of Urology, Keio University School of Medicine, Tokyo, Japan
[3]Department of Nephrology, Tokyo Medical University, Tokyo, Japan

* **Correspondence:** Norihisa Miki, Department of Mechanical Engineering, Keio University, 3-14-1 Hiyoshi, Yokohama, 224-8522, Japan.
miki@mech.keio.ac.jp

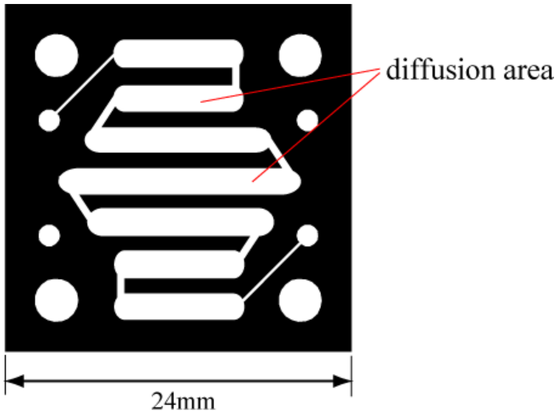

Supplementary Figure 1. Detailed design of the micro channels. The diffusion areas are 2 mm in width and 72 mm in total length (from the top lines: 8, 8, 12, 16, 12, 8, 8 mm in length). The narrow channels that connect the diffusion areas have the width of 1 mm. The channels 0.2 mm in width and 7 mm in length are designed to connect the diffusion areas with the inlet and the outlet. The channel height is 200 μm. The channels are designed to have a sufficient volumetric flow rate at human blood pressure [Gu and Miki, 2009].

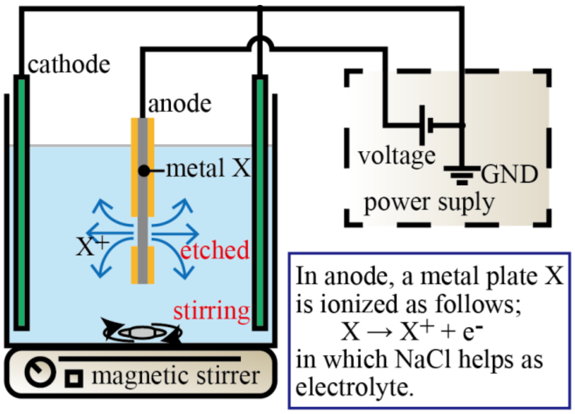

Supplementary Figure 2. Electrolytic etching. The exposed areas of the metal that works as the anode is etched in the electrolytic solution. Negative photoresist SU-8 3050 is patterned on a plate of SUS316L 200 μm in thickness. It is immersed into the electrolytic solution of ethylene glycol, sodium chloride, citric acid and DI water. The voltage of 20 V is applied for 300 minutes to create the channel patterns.

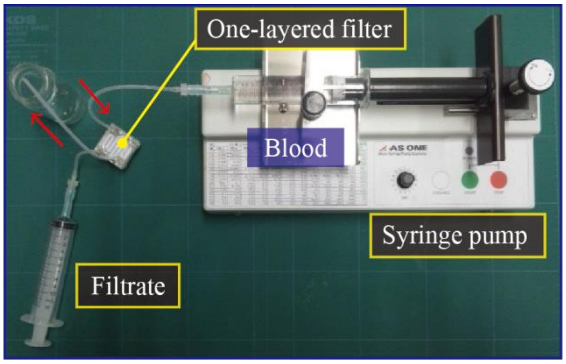

(a)

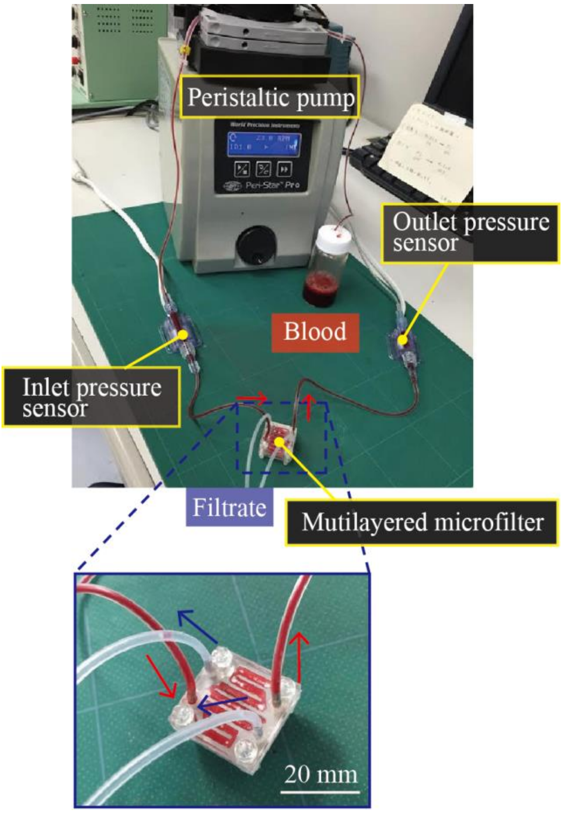

(b)

Supplementary Figure 3 Experimental setup to investigate the water permeability using (a) a single path system and (b) a loop system. (a) A syringe pump was used to introduce whole cow blood into the system to characterize the PES membranes in Table 1. (b) Multi-layered devices required more mass flows of blood than the single-layered device. A peristaltic pump was used to circulate blood to achieve the mass flows.

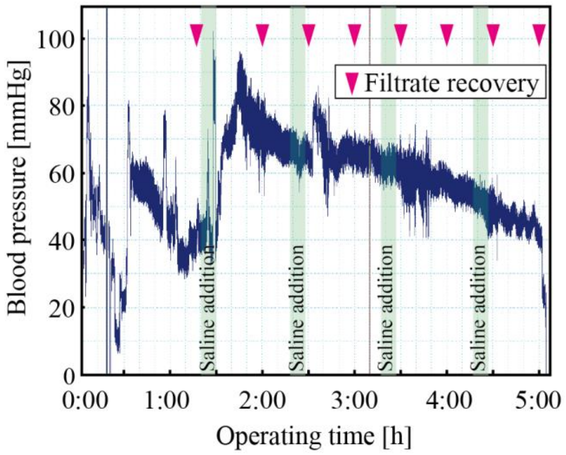

Supplementary Figure 4 Blood pressure of a rat during an *in vivo* experiment. The blood pressure dropped and fluctuated soon after the device was connected because a fair amount of blood was extracted from the body to fill the tube and the device. Saline solution was injected to compensate for the blood pressure drop.
